# An experimental examination of object-directed ritualized action in children across two cultures

**DOI:** 10.1371/journal.pone.0206884

**Published:** 2018-11-28

**Authors:** Rohan Kapitány, Jacqueline T. Davis, Cristine Legare, Mark Nielsen

**Affiliations:** 1 The University of Oxford, Oxford, England; 2 The University of Cambridge, Cambridge, England; 3 The University of Texas at Austin, Austin, United States of America; 4 The University of Queensland, Brisbane, Australia; 5 The University of Johannesburg, Johannesburg, South Africa; Jiangsu Normal University, CHINA

## Abstract

Ritualized actions are common in daily life, and prevalent across cultures. Adults have been shown, under experimental conditions, to treat objects subjected to ritualized action as special and different relative to objects subjected to non-ritualized action. Similarly, children as young as 4, are sensitive to ritualized actions–frequently reproducing such actions at high fidelity. The current cross-cultural experiment attempts to extend existing findings among two culturally distinct groups of children with regard to object-directed rituals. We predicted that children’s preference for a reward would be influenced by ritualized action (but not non-ritualized action). Over two trials we presented children in Australia (N = 93; mean age = 6.03 years, SD = 2.07 years) and Vanuatu (N = 109; mean age = 6.13 years, SD = 1.96 years) with two identical rewards, which was either subjected to ritualized action or non-ritualized action. Contrary to previous findings among adults, ritualized action did not influence children’s preference for a reward. We frame the current results in the context of socially relevant group rituals, and discuss the implications for both wider theory and methods. We conclude with a call for pre-registered replications.

## Introduction

Throughout our lives we acquire a great deal of cultural knowledge, enabling us to navigate our physical and social worlds, and the objects within, often without any awareness of our reliance on this knowledge. The reader is likely effortlessly able to describe what behavior is expected of them at Christmas, at a funeral, at a wedding, or at a birthday. Moreover, this knowledge involves an understanding of certain ritualized objects, such as presents, particular clothes, and the significance of rings. While the specifics of ritual events vary between cultures, all cultures have rituals that make context specific demands [1,2].

Ritual appears to have many and diverse effects. Participating in rituals helps in forming, maintaining and facilitating within-group co-operation [3–10]. And merely observing rituals can influence identity [11], religious belief [12], memory [13,14] and emotion [11]. There is also an argument that engaging in ritual practices emerged relatively early in hominin evolution [15, 16]. Yet less is known about how rituals influence our understanding of *objects* or *artefacts*, despite the fact that rituals frequently require the use of, or are directed toward, them. Early research suggests that that objects—in this case, food—subjected to ritualistic action are perceived as more valuable and tastier [17] and can improve food-related decision making behavior [18]. Similarly, Kapitány & Nielsen (2015, 2016) have shown that ritualistic actions appear to make otherwise physically identical objects more special and more desirable. Further, while there is evidence that children are sensitive to ritualistic cues in varied social contexts, there is no literature on how children understand and relate to ritualized objects. Our aim here is to conceptually replicate and extend the findings of Kapitány & Nielsen (2015; 2016) among children, and to do so among two culturally distinct populations.

Ritualistic actions are actions which are causally opaque and goal demoted, due to repetition, redundancy, formality, and/or stereotypy of action [19–25]—features which disrupt and obscure causal interpretations [14,26–28]. One framework for understanding the relationship between rituals and objects is the ritual stance [29], which describes the tendency of both children and adults to interpret ritualized action as socially normative and informative [9,30]. This is due to the fact that the actions are deliberately and willfully performed, while not being instrumentally relevant. The ritual stance suggests that ritualized actions are understood as motivated by social concerns [30,31], and the framework successfully predicts judicious, high-fidelity imitation of such actions [32,33]. The alternative to the ritual stance is the ‘instrumental stance’, in which non-ritualized, or ‘ordinary’ actions, are interpreted as functional. Such actions are readily understood causally, and the intention of the actor is easily understood as corresponding with bringing about the observed effect.

Previously, Kapitány & Nielsen (2015; 2016) demonstrated that the ritual stance can successfully predict social and normative evaluation of objects which have been subjected to ritual. Their work involved developing an array of matched, ritualized or non-ritualized, actions toward a specific object [a glass filled with liquid]. A non-ritualized action was a typical and ordinary cleaning action (e.g., rubbing an empty glass with a cloth, scratching grit off a glass with one’s thumb), while a ritualized action involved the same movements and motor features, but was performed at a remove from the object so as to deny any possible physical-causal link. That is, a circle was drawn in the air near to the rim of the glass, without making contact with the glass. These two kinds of actions were matched at the level of motor features, repetition, willfulness, and duration, and yet the ritualized action was causally opaque and goal demoted due to the absence of an inferrable outcome (videos of these actions can be seen here: https://osf.io/qtm5r/). Over three experiments (conducted online), Kapitány & Nielsen (2015, 2016) presented over 1,300 adults with videos of ritualized or non-ritualized actions directed toward a specific object among an array of identical objects. They found that participants reliably attributed ‘specialness’ toward the ritualized object, as well as having increased preference for the object. Interestingly, neither kind of action predicted perceptions of a physical change in the object itself. In one study, they found that even when the actions were associated with negative out-groups, specialness and preference were still increased by ritualistic action. This is consistent with the ritual stance: such actions are understood as being socially important.

Here, we aimed to replicate and extend the work of Kapitány & Nielsen (2015, 2016) by examining their paradigm among children in diverse cultural contexts. There is evidence that children perceive ritualized action as. [socially informative and normative [29,32–37]. And that this sensitivity is apparent in multiple cultures [38–40]. Typically, children’s sensitivity to ritual cues is measured by examining either the fidelity of their copying or the degree to which children bond to their group [6,41,42]. For example, Watson-Jones et al. [43] presented children (aged 3–6 years) with adult-models demonstrating behaviors on a peg-and-block apparatus that were either ordinary / non-ritualized (satisfying the affordances of the apparatus; i.e., blocks were placed on specific pegs via ritualistic action), or were ritualistic (the ritualized action, while deliberate, did not alter the configuration of the apparatus from the start-state to the end-state). When the sequence was maximally ritualized and goal demoted children imitated at higher rates than when it was not, and provided explanations for their own behavior that appealed to convention. This effect was conceptually replicated among children aged 4–6 years [30].

In other work, when children (aged 3–6 years) were shown ritualized actions that were presented by one actor twice, two actors acting sequentially, or multiple actors acting simultaneously, the fidelity of a child’s imitation of redundant actions in the latter condition was greater than in the former conditions [35–29], though notably, even in the least social condition, children still copied well above null rates. Finally, Wilks, Kapitany, Nielsen found a sensitivity to ritualized action: they showed children (aged 4–6 years) individual models, and group-associated models, performing actions on a puzzle box. The model performed either instrumental or ritualistic actions, which were enhanced when the models either successfully retrieved the reward from inside the box, actively eschewed retrieving the available reward, or were not shown doing either (in the latter case, the video stimuli cut out after the model made the reward available. In the former instances, once the reward was made available, the video showed the model retrieving, or ignoring, the reward respectively). When the video cut out, children were not more likely to copy than when the model explicitly retrieved the reward. However, children were over three times more likely to copy ritualistic actions when the group-associated model actively eschewed the reward, than they were to copy the individual who actively retrieved the reward. Children, thus, appear sensitive and responsive to ritualized action, even when such actions come at a material opportunity cost. The point here is that children (typically between the ages of 4 to 6) tend to be sensitive to causally opaque and goal demoted actions–copying them when given the opportunity [44]. Moreover, such imitative tendencies regarding causally opaque actions have been demonstrated in a diversity of ‘non-WEIRD’ cultures [38–40].

While ritualized action may generate in observers a normative, socially oriented understanding of the action, which can be meaningfully measured by imitative fidelity, not all rituals require those who observe them to subsequently re-create them. Examples include a priest consecrating wine, a flag being lowered to half-staff, the exchange of rings at a wedding, or the decoration and presentation of a birthday cake. After the initial ritualized action, the same action does not need to be performed again, yet one may need to adapt their behavior in response. Children also learn that certain ritualized objects are special, which in turn require different kinds of treatment. Holy books are not like ordinary books, Grandma’s cremation urn is different from the other vases on the shelf, and Mum’s engagement/wedding rings are different from her other rings. Here we aim to investigate a child’s behavior and relationship with objects that have been subjected to ritualized action, in a manner distinct from prior imitative, affiliative, contexts.

One study that may shed light on how children understand the relationship between rituals and objects. 107 Israeli children were interviewed (aged 4–9 years) on their understanding of the Birthday Party [45]. When asked which ‘customs’ of the birthday party were essential, 21 (20%) declared that *every* custom was essential and 51 (48%) declared that *some* customs were essential. Only 15 (14%) of children indicated that there were no essential features of the ritual. Unsurprisingly, all but three (of 15) who said no feature was essential were from the oldest age bracket. When asked ‘*Why do we celebrate a Birthday*?’ the modal response (40%) was that the Birthday Party increases one’s age, while 28% said that it was used to determine and declare one’s age. And yet adults, who host such parties for their children and their peers, do not hold these same beliefs. Rather, adults see the acts as social and normative. Children lose their ritual-essentialist beliefs as they get older, yet it seems as though children younger than 9-years of age infer that ritualized objects are not simply important, but essential.

In the current research, we aimed to investigate the nature of object-directed ritualized action, in an attempt to replicate and extend work in adults. We looked explicitly at the preference children demonstrated for objects when presented with identical choices, which vary only on whether they were subjected to ritualized- or non-ritualized action. The question under consideration is–given that adults appear to represent ritualized objects as physically similar, but special and desirable–whether or not children prefer to select the ritualized objects over non-ritualized alternative. We examined the behavior of children across a range of ages, by adapting the protocol of Kapitány & Nielsen (2015; 2016). In addition to this, we attempted to determine how robust the effect was to cultural influences (if the anticipated effect exists). Psychological research [46], and developmental research in particular [47,48], tends to examine populations that are not generally representative of humanity (heuristically, participants are commonly *‘Western*, *Educated*, *Industrialized*, *Rich*, *and Democratic*’; i.e., ‘WEIRD’), which may produce a skewed and unrepresentative understanding of human psychology and behavior. Thus, we examined two distinct child populations: one in Brisbane, Australia, and one on Tanna Island, in The Republic of Vanuatu. These populations were selected on the basis of the relative independence from one another, and their relative convenience to the research team. Conclusions drawn from this data, which cover a wide range of ages, in two distinct cultures, should allow for stronger inferences with regard to our central questions than if only one culture was evaluated.

In the present experiment we modified and simplified a protocol used in prior work that explicitly examined behavior with regard to object-directed ritualized action [19,34]. Here, children observed the experimenter performing either a ritualized or a non-ritualized action on one of two bowls, each of which contained an identical reward. After the presentation, children were given the opportunity to select a reward from one of the two bowls. This process was then repeated a second time. Additionally, we minimized language cues in order to increase the focus on the action. The primary difference between the present protocol, and prior protocols, is that this was performed *in situ*, rather than online, and was conducted among children rather than adults.

We predicted that *1)* there would be a main effect of action-type, such that ritualized action would produce greater preference for the rewards in acted-upon bowls than non-ritualized action, *2)* that there would be a main effect of age, such that older children would be more likely to select the acted-upon bowl independent of condition, and *3)* that there would be an interaction term such that as children increased in age they would increasingly prefer objects subjected to ritualized action. As there were no *a priori* reasons to expect cultural differences between populations we made no specific predictions, but we included this as a predictor in our analyses for the sake of analytic rigor.

### Vanuatu

The Republic of Vanuatu is located in the South Pacific, and is a Melanesian culture. Tanna Island, one of many islands that make up Vanuatu, and the island on which this research was conducted, is north of New Zealand and south-east of Papua New Guinea (approximately halfway between the two nations) and several hours flight from Brisbane, Australia. Vanuatu was selected as a good candidate site for this research, not simply because of its status as a ‘non-WEIRD’ culture, but because ritual is a central aspect of Ni-Van life [49,50]. Moreover, Ni-Van populations have been quite well studied. Child populations on Tanna Island have been involved in a number of research projects, some of which specifically relate to ritual cognition. For example, Rybanska et al. (2017) conducted an intervention study among Ni-Van children examining the influence of ritualistic behavior on their ability to delay gratification. They found that participation in ritualistic activities (compared to instrumental activities and a control condition) improved executive function and performance on a delayed gratification task. The authors speculated that children accustomed to ritual generally may be more sensitive to novel rituals, but allowed for the possibility that ritual sensitivity may be somewhat independent of cultural context. Their findings indicate no observable difference in behavior between the Ni-Van children and a sample of Slovakian children. In support of the cognition-over-culture interpretation, Clegg & Legare (2016a) and Rybanska et al. (2017), using an established necklace-making ritual protocol [29,41,51], found that coastal Ni-Van children imitated ritualistic actions at higher rates than instrumental actions, and at rates comparable to that of a US sample. In the present study we do not make any specific cultural predictions (as we have no evidence to support them). We include the Ni-van culture primarily in an attempt to create more inclusive and generalizable conclusions.

## Method

### Participants

#### Brisbane, Australia

Brisbane is a large, developed, English speaking city with compulsory free education (for children aged 5 years and older). The population of Brisbane is around 2.35 million people, and is typical exemplar of a ‘WEIRD’ society. Testing was conducted in Brisbane’s `*Sciencentre’*, a large, public science museum located very near the central business district. We recruited a convenience sample of 93 children aged between 2–13 years as they were entering or leaving the centre (mean age = 6.03 years, SD = 2.07 years; 2 values missing). Parents/Guardians provided consent via electronic and verbal means.

#### Ikunala and Yakel village, Tanna Island, The Republic of Vanuatu

The second and third locations were on the island of Tanna in Vanuatu. Specifically, we tested children in the ‘*Kastom’* villages of Ikunala and Yakel. Here, indigenous Melanesians live their lives according to ‘*Kastom’* (a traditional set of beliefs and practices). Ikunala is located inland from the coastal village of Lenakel.

With the consent of village elders, we attempted to exhaustively include all children from the village in our study. Additionally, a number of children/families from neighboring Kastom villages travelled to participate (our sample, then, is the upper limit of what was possible to collect at that time). We did not observe any children speaking English (though some adults spoke rudimentary English, and a great number spoke Bislama—an English-creole and one of the official languages of Vanuatu). Children in Ikunala do not receive a formal education. Unlike Yakel, Ikunala does not typically allow visitors into their villages without a clear purpose, and it does not generate any revenue from allowing tourists or outsiders (such as film crews) to visit. Residents of Ikunala retain a very traditional way of life, and western influence is minimal.

Yakel is a Kastom village, unrelated to Ikunala, located further south. Similar to Ikunala, children do not routinely receive formal education, and–to the best of our knowledge–do not speak English. The primary difference between these locations is that Yakel is more widely known, and is more accepting of visitors in exchange for gifts and–specifically—money. We provided both Ikunala and Yakel with gifts of food (e.g., coconuts, canned fish) and Kava in exchange for their participation (Yakel also received some money paid to the Chief). Both villages speak dialects of the ‘Navhal’ language.

The ages of all children (at both locations; N = 109) were measured in whole years, and ranged from 3–9 years (mean age = 6.13 years, SD = 1.96 years; 2 values missing). It is worth noting that Kastom communities in Vanuatu do not record their ages or birthdays. Where possible, best estimates of children’s ages were given by members of the translation team who were personally familiar with the children.

The University of Queensland Behavioral & Social Sciences Ethical Review Committee (BSSERC) provided ethical approval for this project (#2010001558), which included obtaining oral consent from village elders and family member in Vanuatu. Furthermore, we received approval from the Vanuatu National Cultural Council and the Vanuatu Cultural Centre in order to conduct this research.

### Procedure

The present experiment was a between-subjects design, in which children observed an experimenter acting upon one of two bowls, where each bowl contained an identical small reward (a sticker). The actions the experimenter performed were either ritualized (causally opaque and goal demoted) or non-ritualized in nature. Two actions were adapted for use with children based on prior work [19,34]. One action employed a cloth: in the non-ritualized condition the cloth was used to clean the bowl, while in the ritualized condition the cloth was waved toward the bowl without making contact. The alternate action involved only the researcher’s hand: in the non-ritualized condition the researcher picked up the bowl and cleaned/rubbed/scratched at it with his fingers as if moving grit or a stain. In the ritualized condition, the hands and fingers were moved in a similar manner, slightly exaggerated, and in such a way that the action did not make contact with the bowl. In this way, the ritualized action was goal demoted (the intention of the actor was unclear) and causally opaque (how the actions brought about a specific effect was not apparent). The non-ritualized action had similar motor-features, but were causally transparent and goal apparent. All actions were performed toward a bowl for several seconds, before the bowl was given a half turn, and the actions repeated on an alternate face.

In Brisbane, the experimenter approached families with children in the *Sciencentre* and invited them to participate in the research. Children sat at a table across from the experimenter. The experimenter then showed the child two identical stickers, and placed one into each of two identical bowls in front of the child. According to condition, the experimenter performed an action toward one of the bowls and, afterwards, told the child that they could have whichever sticker they wanted. Both the action [cloth/hand], and bowl location [left/right] were randomized on the first trial and reversed on the second (such that a child did not see the same action twice, nor were the actions performed on a bowl at the same location twice). After the child chose in the first presentation, the bowls were stacked and the remaining sticker removed. The process was then repeated. In both presentations, at the point of choice, the experimenter withdrew from the interaction to avoid influencing the child’s choice through gaze or gesture. After the process was complete children were then asked their age and date of birth. Only a single experimenter was involved during the testing phase among Brisbane children.

On Tanna Island, the process was identical to that employed in Brisbane, except that the speaking role was carried out by translators (while the lead author executed the actions). Additionally, village chiefs and heads of families gave collective consent, and all age appropriate children in the village participated. Translators said to the child in their native language *“Look at the man* [n.b. the lead author], *he’s got two stickers*. *He’s going to do some actions*, *then you can choose one of the stickers to have''*. After the actions were performed the experimenter said *“You can choose whichever sticker you like''*, which was translated for the child. Translators were trained to never indicate to the child a specific bowl, either through gaze, gesture, or words. Instructions were back-translated into English to the lead author’s satisfaction prior to data collection.

### Coding and measurement

A single point was scored if a child selected the acted-upon bowl in each trial. Age was measured in whole years and treated as a continuous variable. We did this because we had no specific non-linear developmental hypotheses, and thus, had no reason to model the data with age binned into specific categories.

## Results

Table 1 shows the distribution of participants across conditions and regions. Among Ni-Van children, 54 (49.5%) of participants were in the non-ritual condition, and 55 (50.5%) were in the ritual condition; Among Australian children, 48 (51.6%) were in the non-ritual condition, and 45 (48.4%) were in the ritual condition. Table 2 shows the distribution of the dependent variable by condition and age.

**Table 1:** β and Odds Ratios for the logistic regression for both trials.

**Trial 1**				
		**95%CI**		
**Predictors**	**β (SE)**	**Lower**	**Odds Ratio**	**Upper**
**Constant**	.975 (.706)	.68	2.65	10.97
**Condition**	.684 (.974)	.29	1.98	13.63
**Age**	-.128 (.105)	.71	.88	1.08
**Condition:Age**	-.072 (.152)	.69	.93	1.25
**Nation**	-.411 (.295)	.37	.66	1.18
**Trial 2**				
**Predictors**	**β (SE)**	**Lower**	**Odds Ratio**	**Upper**
**Constant**	.172 (.689)	.31	1.189	4.66
**Condition**	.345 (.938)	.22	1.41	8.97
**Age**	-.036 (.102)	.79	.96	1.18
**Condition:Age**	-.029 (.146)	.73	.97	1.29
**Nation**	.236 (.291)	.72	1.27	2.25

**Table 2:** The distribution of participants across conditions, and regions, as a function of age.

	Non-Ritual Condition		Ritual Condition		
	Australia	Vanuatu	Australia	Vanuatu	Totals
**2–3 years**	1	0	0	0	1
**3–4 years**	8	13	10	16	47
**5–6 years**	25	16	21	21	83
**7–9 years**	11	23	8	18	60
**> = 10 years**	3	0	4	0	7
**missing**	0	2	2	0	4
**Total**	102		98		202

We combined scores across both trials into a single score. This aggregate score had three levels (0 = always avoid [acted upon object], 1 = mixed preference, 2 = always prefer). We specified an ordinal logistic regression model (using the MASS model on R; [52], with Condition (Hypothesis 1), Age (Hypothesis 2), and an interaction between Condition and Age (Hypothesis 3) entered as predictors. We included Nation as a predictor for completeness. However, in so doing we determined that the test of parallel lines was significant, χ^2^(4) = 10.356, *p* = .035. This indicates that the ‘distance’ between outcomes ‘0’ and ‘1’, and ‘1’ and ‘2’ were not equivalent. Thus, we conducted two separate binomial logistic regressions (the comparatively large differences between constants, particularly relative to the predictors, indicates the nature of violated assumption; see Table 1).

We specified a logistic regression model for both trials, with Condition (Hypothesis 1), Age (Hypothesis 2), and an interaction between Condition and Age (Hypothesis 3) entered as predictors. We included Nation as a predictor for completeness. Regarding the first trial, our model did not provide good fit, χ^2^(4) = 7.84, *p* = .010. None of the predictors contributed to the model (Condition, *p* = .167; Age, *p* = .221, Condition:Age, *p* = .638; Nation, *p* = .163). Regarding our second trial, our model did not provide good fit, χ^2^(4) = 1.53, *p* = .821. None of the predictors contributed to the model (Condition, *p* = .713; Age, *p* = .724, Condition:Age, *p* = .840; Nation, *p* = .416). Table 2 lists β and Odds Ratios (OR) for both trials, Table 3 record raw behavior across both trials by condition and age. The reference category for the OR should b interpreted as indicating the likelihood that a participant selected the acted-upon object over the non-acted-upon objects. Thus, In trial 1, condition the OR indicates that children were 1.98 times more likely to select the acted-upon object over alternatives (note, however, that confidence intervals cross 1.00; this effect is not significant).

**Table 3:** Behavior across both trials by condition and age.

	Non-Ritual Condition			Ritual Condition		
	Always Avoid	Mixed Preference	Always Prefer	Always Avoid	Mixed Preference	Always Prefer
2 years	0	1	0	0	0	0
3–4 years	7	4	10	7	7	12
5–6 years	14	16	11	10	13	19
7–9 years	12	11	11	11	8	7
> = 10 years	1	1	1	0	3	1
Missing	2	0	**0**	0	2	0
**Total**	**36**	**33**	**33**	**28**	**33**	**39**

Note: ‘Always Avoid’ describes the tendency for a given child to always select the control bowl; ‘Always Prefer’ describes the tendency to always prefer the test bowl; ‘Mixed Preference’ describes the tendency to switch decisions.

## Discussion

Our central question was whether children would prefer to select a ritualized object over a non-ritualised alternative. Past research has shown that adults view objects subjected to ritualized action as more special and desirable than objects subjected to non-ritualized action, or to no action at all. We failed to find evidence that children were influenced by ritualized actions with regard to object choice. We expected a linear age effect, where increasing age would lead to increasing preference for acted-up upon objects, and specifically ritualized objects. We observed no effect of age for either trial. Moreover, we failed to find evidence for condition or an interaction. Thus, we failed to find support for our hypotheses. We find no effect of nation/cultural group; our data are consistent across two distinct cultural groups: Ni-Van children in *kastom* villages in Vanuatu, and children in a large industrialized city in Australia.

Prior research (outlined in the introduction) has shown that children aged between 3 and 6 years are sensitive to ritual cues, copying at high fidelity, and doing so for what appears to be conventional and social reasons. The majority of our sample fell into this age range. Moreover, data from Israel suggest that children in this age range interpret the ritualized objects in social contexts as essentially necessary, but that this belief declines as children age beyond 6 years. This present work attempted to replicate and extend work conducted by [19,34], who observed the effect of preference in samples that were considerably older (where the youngest sample had a mean age of 19.76 years; SD = 3.20). Herein, we observed no [apparent] sensitivity to object-directed novel rituals.

Is it possible, then, that object directed rituals are different in some way from social- or group-oriented rituals? Children, like those we tested in Brisbane, have an empirical history of being sensitive to ritualized action, where such actions increase preference for in-groups, imitation fidelity, and functional fixedness [29,31,37,51,53]. Admittedly, whether children were sensitive to object-directed rituals was an open question, yet it seems reasonably parsimonious to assume that object- and group-related ritual cognitions are related—indeed, our operationalization of ritualized action was consistent with the majority of similar work. Our ritualized actions were repetitive, redundant, goal demoted, and causally opaque [19–25], and also maintained start-end state equivalency [43]. While less research has been conducted in Vanuatu (relative to ‘WEIRD’ locations), at least two published papers indicate a level of ritual sensitivity among children comparable to ‘typical’ samples (see: 41,50). Thus, if children are sensitive to group-related ritual actions, but not to to object-directed ritual action, we must entertain the idea that object-directed rituals are dissociable from other kinds of rituals that share the same features and definitional qualities.

A null finding, of course, is not necessarily evidence for the null, but rather a lack of evidence for the test hypotheses [54]. This is not to shift the goal-posts nor declare success in the face of failure (or, at least, the absence of success), it is simply to describe the data as inconclusive. We have identified three key differences between the existing literature and the present findings that are worth discussing in light of these results. 1) This work varies from prior work, in that prior work (Kapitány & Nielsen, 2015; 2016) administered their manipulation using video stimuli to participants over the internet. Thus, there may be a meaningful difference between *in situ* and *in vivo* testing with respect to rituals; 2) Due to the cross-cultural nature of this research, we attempted to minimise the use of language in both contexts. This decision is somewhat at odds with typical methodologies used in this field of research, which tends to use ‘conventional’ language in association with ritualised action (this point will be elaborated below); and 3) it is possible that features of the present experiment—including the status/identity of the individual performing the ritual actions, and the target-objects of the ritual—were inappropriate (to be discussed below). A fourth and final possibility is that prior work amongst adults examined the behavior in over 1,300 adults, while the present research is limited to only 203 children. We note that the [real] effect (if one exists) may be too small to be detected (while noting that 200 participants is sufficient to detect an effect associated with an OR of 1.5 with 90% confidence).

Research into the validity of online samples has shown that they do not vary considerably from *in situ* samples [55–57]. Indeed, Kapitány & Nielsen (2015; 2016)—who made their observations using online surveys and video stimuli—wrote of their original finding that *“[Future research] under lab conditions employing face-to-face testing will resolve the true extent of this phenomenon*.*”* (p. 27). It seems counterintuitive that the effect of ritual is most apparent in virtual circumstances, but absent in the ‘real world’ (where rituals are used every day). During the production of this manuscript, Kapitány and Nielsen attempted to behaviorally replicate their prior findings (2015; 2016) among adults in a behavioral context. Similar to the present study, participants were exposed to either ritual or non-ritual actions directed towards focal objects within an array of otherwise identical objects. Participants saw one presentation in which the action was either ritualized or not, and selected their preferred object. Immediately following this, participants saw the same kind of action as in the first presentation, but it was accompanied with a claim the action was a blessing or a curse. Participants then completed a battery of measures administered on a computer. Among 101 undergraduates, there was no effect of action-type on a participant’s preference for an object, nor was there an effect on self-reported specialness. That is, Kapitány & Nielsen (in press) failed to replicate, in a behavioral context, their own earlier work. These findings seem qualitatively similar to the data presented here. It is not necessarily clear whether the behavioral aspect explains this difference, or other features are at play (as described below).

The second possibility is that ritual studies on children frequently employ conventional language (see: [29,51,58,59]. Rybanska et al. studied the relationship between ritualized action and self-control in Vanuatu, included the following statement alongside demonstration of the ritual actions: “*it has always been done this way*” or *“those are the rules and they must be followed*”. This was done in order to “*encourage interpretation of the actions as conventional rather than instrumenta*l” (p. 4). Indeed, in the original work by Kapitány and Nielsen, cues describing the actions as belonging to social groups (2015) or serving specific magical purposes (i.e., to bless or curse; 2016) were used. Such language was deliberately avoided here, on the premise that the actions alone would sufficiently arouse responses consistent with the ritual stance. Notably, Kapitány and Nielsen (*in press*) actively avoided using conventional cues when presenting adults with similarly ritualized and non-ritualized actions, and they too failed to find predicted responses. The ritual actions employed in the present study are conceptually and practically similar to ritual actions used in a wide range of studies. Thus, it is possible that group-related and conventional language cues are the most salient cues to ritualized action, and may also account for some unknown amount of explained variance.

The third possibility relates to the theoretical structure of ritual. [60] suggest that there is a key set of necessary features of rituals: agents, instrument, acts, and patients. The ‘agent’ of a ritual is the individual performing the act, and there is some evidence that certain kinds of agents are better than others (i.e., priests vs. laymen; [61]. Instruments are the tools required to execute the action. Not all rituals require instruments, but, again, some evidence exists that some objects are better than others [61]. Next, the nature of the actions performed is important. Evidence shows that the number of steps, repetition of actions, and specificity in time, increase one’s interpretation that ritualized actions will be successful [23]. Finally, a ‘patient’ is the subject of the ritual. In the present case, the patient was a bowl which contained a sticker. In Kapitány & Nielsen (in press), the patient was also an artefact. However, earlier work has shown that when the patient is *food*, people consider ritualized actions to have considerable influence—ritualized food is more valuable and tasty [17] and more special and desirable [19,34]. It is possible that the null effects observed here relate to the nature of the ritual patient: artefacts are inherently less ritualizable than foodstuffs. This, of course, is an empirical question worthy of investigation, but one outside the scope of the present study.

There are two additional elements that relate to the structure of the ritual action itself. The first relates to the agent (i.e., the researcher) performing the actions, while the second is in regard to the subject of the ritual. In the first instance, the agent may not have been perceived as being neither a relevant model for ritual behavior, and/or incapable of bring about the ritual’s effect. (Though it is worth noting that the same researcher was present in the experiments, Kapitány & Nielsen 2015;2016, in which the effect was found. And also that these attribution may not necessarily be cultural in nature, as the Australian and Ni-van children behaved in a manner that was not discriminable). With regard to the subject, we note here that the ritual actions were performed more directly in relation to the ritualized *vessel*, rather than the on the prize itself. It is possible that this distinction washed out the apparent effect. While this is a potential confound, one which is unresolvable with the present data, we suggest it’s unlikely to account for the [absence of the] effect. Mental contagion, as documented by authors such as Nemeroff and Rozin [62][63], have shown that essentialist and ritualistic qualities can be ‘caught’ or transmitted between objects via contact and action. It is difficult to envisage how a ritualized action on the vessel was not also cuing some information for our participants regarding what would subsequently be contained *in* the vessel. Again, we do not rule this possibility out, but do not believe it is a particularly powerful explanation of the null results.

So what can we say about the present findings? We know that when a ritual is related to group belonging, or other explicit social conventions, children are typically quite sensitive. Not only do they tend to universally imitate ritualized action at higher rates than control actions [38, 44, 64, 65], but that such actions appear to influence basic cognitive processes, such as executive function [50]. Children are not only able to identify when actions are redundant (i.e., ritualistic; [66], they can selectively omit their reproduction when doing so is not socially useful or explicable [33]. Yet children will normatively enforce such actions under social circumstances [32,36] - consistent with the ritual stance. Further, we know that until a child is 9-years old, they believe that certain rituals (like the birthday party) effect change in the world, and *essentially require* ritualized action [45].

Here we fail to find evidence of a preference for objects subjected to ritualized action among two diverse populations of children. While the ritual stance is a powerful predictor of a child’s behavior with regard to group-related outcomes, it may be the case that inferring social information from object-directed ritualized action (for children, and possibly adults) is more difficult, or more complicated, than previously anticipated. Particularly when linguistic cues associated with group-belonging are absent—children may not have a tendency to generate such inferences about novel objects. If this is the case, it may be that the inferential potential of novel ritualized action towards objects is *learned* over the course of one’s life—thus explaining the pattern of adult-data in Kapitány & Nielsen (*in press*). That is, the null result of Kapitány & Nielsen (*in press*) in their adult behavior experiment—as with the present experiment—may be explained by the fact that the ritualized actions were novel, and the participants had not learned the cultural and symbolic value of the actions. This is speculative, however, as our data do not provide evidence for this claim. We present this work to help contextualise previous findings, and to avoid ‘file-drawing’ this research to protect previous publication. To determine whether this null result present here is truly inferentially meaningful, or the product of sampling and/or chance, pre-registered replication attempts are now required. To this end, the authors have made all data available on the Open Science Framework (https://osf.io/qtm5r/), and welcome correspondence and replication attempts.
